# Evaluation of the Bruker Biotyper Matrix-Assisted Laser Desorption/Ionization Time-of-Flight Mass Spectrometry System for Identification of *Aspergillus* Species Directly from Growth on Solid Agar Media

**DOI:** 10.3389/fmicb.2017.01209

**Published:** 2017-06-29

**Authors:** Ying Li, He Wang, Yu-Pei Zhao, Ying-Chun Xu, Po-Ren Hsueh

**Affiliations:** ^1^Department of Clinical Laboratory, Peking Union Medical College Hospital, Chinese Academy of Medical SciencesBeijing, China; ^2^Graduate School, Peking Union Medical College, Chinese Academy of Medical SciencesBeijing, China; ^3^Department of General Surgery, Peking Union Medical College Hospital, Chinese Academy of Medical SciencesBeijing, China; ^4^Departments of Laboratory Medicine and Internal Medicine, National Taiwan University Hospital, National Taiwan University College of MedicineTaipei, Taiwan

**Keywords:** matrix-assisted laser desorption/ionization time-of-flight mass spectrometry system, Bruker Biotyper MALDI-TOF MS, *Aspergillus* species, solid agar media, sequence analysis

## Abstract

We evaluated the accuracy of the Bruker Biotyper matrix-assisted laser desorption/ionization-time of flight mass spectrometry (MALDI-TOF MS) system at identifying clinical isolates of *Aspergillus* species that were grown on agar media. A total of 381 non-duplicate *Aspergillus* isolates representing 21 different *Aspergillus* species identified by molecular analysis were included in this study. The Bruker Biotyper MALDI-TOF MS system was able to identify 30.2% (115/381) of the isolates to the species level (score values of ≥2.000) and 49.3% to the genus level (score values of 1.700–1.999). When the identification cutoff value was lowered from ≥2.000 to ≥1.700, the species-level identification rate increased to 79.5% with a slight rise of false identification from 2.6 to 5.0%. From another aspect, a correct species-level identification rate of 89% could be reached by the Bruker Biotyper MALDI-TOF MS system regardless of the score values obtained. The Bruker Biotyper MALDI-TOF MS system had a moderate performance in identification of *Aspergillus* directly inoculated on solid agar media. Continued expansion of the Bruker Biotyper MALDI-TOF MS database and adoption of alternative cutoff values for interpretation are required to improve the performance of the system for identifying highly diverse species of clinically encountered *Aspergillus* isolates.

## Introduction

Infections due to *Aspergillus* are associated with high morbidity and mortality rates among hospitalized patients, especially for those who are immunocompromised or suffer from serious underlying diseases (Brown et al., 2012; Liu et al., 2015; Taccone et al., 2015; Wang et al., 2016b). *Aspergillus* is the second most common pathogen causing invasive fungal disease (IFD) after *Candida* species and the number of *Aspergillus*-related infections has been recently rising gradually (Liao et al., 2013). Traditionally, identification of *Aspergillus* in clinical mycology laboratories is based on phenotypic traits such as colony structure, color, and growth rate as well as on micromorphological profiles such as the shape of conidia, spores, and mycelial structures (Ciardo et al., 2007). Conventional identification methods, however, have some significant drawbacks. First, they are time-consuming to perform and are associated with long turnaround times for results (5–14 days). Second, some phylogenetically related *Aspergillus* species share the same or similar structural features when grown under laboratory conditions but may have different pathogenicity and drug susceptibility profiles, which can prevent identification or even lead to misidentification. Third, some *Aspergillus* characteristics are unstable, sometimes manifesting atypically with slow sporulation and aberrant conidiophore formation. As a result, experienced personnel are needed to correctly identify *Aspergillus* in mycology laboratories, which, unfortunately, is uncommon in many primary hospitals, particularly in China (Balajee et al., 2009; Ciardo et al., 2010; Pfaller et al., 2012; Wang et al., 2016a). Nucleotide sequencing of internal transcribed spacer (ITS) regions of ribosomal DNA and beta-tubulin or calmodulin genes is an alternative to conventional methods of *Aspergillus* identification, especially for isolates with unusual phenotypic profiles and for rare *Aspergillus* species (Balajee et al., 2007; Samson et al., 2014); however, this approach is fastidious and not convenient for rapid identification in clinical mycology laboratories (Hsiue et al., 2010; Lamoth, 2016).

Matrix-assisted laser desorption/ionization-time of flight mass spectrometry (MALDI-TOF MS), which is now widely used in clinical microbiology laboratories, can rapidly and accurately identify different species of bacteria and yeasts (Singhal et al., 2015; Cassagne et al., 2016). However, the use of MALDI-TOF MS for identifying clinical molds, especially *Aspergillus*, is restricted, mainly because samples require extensive preparation to achieve good quality mass spectra and the limited fungal coverage in commercial databases (Sanguinetti and Posteraro, 2014; Cassagne et al., 2016). Several studies have reported that the use of in-house databases for the Bruker MALDI-TOF MS biotyper (Bruker Daltonik, Germany) covering mainly the genus of *Aspergillus* can improve the accuracy of *Aspergillus* identification to some degree (Alanio et al., 2011; De Carolis et al., 2012; Lau et al., 2013; Ranque et al., 2014; Schulthess et al., 2014). However, the species coverage of most of these in-house fungus databases is limited, and most of them are neither publicly nor commercially available (Schulthess et al., 2014).

In this study, we evaluated the commercially available fungi database (Filamentous Fungi Library 1.0) developed for the Bruker Biotyper MALDI-TOF MS system for identification of clinical isolates of *Aspergillus* grown directly on solid agar media.

## Materials and methods

### *Aspergillus* isolates

A total of 381 isolates of *Aspergillus* isolated from various clinical specimens (respiratory tract, wound swabs, urine, pus, biopsy specimens, and cerebrospinal fluids) were collected at National Taiwan University Hospital (NTUH, *n* = 198) in Taiwan and Peking Union Medical College Hospital (PUMCH, *n* = 183) in mainland China from January 2014 to December 2015 (Table 1). These isolates were initially identified to the species or genus level based on conventional phenotypic identification methods in the clinical mycology laboratories at NTUH and PUMCH.

**Table 1:** Identification of 381 clinical isolates of *Aspergillus* species by the Bruker Biotyper MALDI-TOF MS system and analysis with reference to identification results by molecular methods.

***Aspergillus* species**	**No. of isolates**	**No. (%) of isolates identified by the Bruker Biotyper MALDI-TOF MS in each indicated range of score values**			**No. (%) of isolates with identification results in each indicated range of score values with reference to identification results by molecular methods**					**No. of references spectra in the Bruker database**
		**≥2.000**	**1.700–1.999**	**≤1.699**	**Correct identification to species level**				**Only correct identification to genus level**	
					**≥2.000**	**1.700–1.999**	**≤1.699**	**Subtotal**		
***ASPERGILLUS*** **SPECIES COMMONLY ENCOUNTERED (*****n*** **≥ 10)**										
*A. fumigatus*	107	18 (16.8)	80 (74.8)	9 (8.4)	18 (16.8)	80 (74.8)	8 (7.5)	106 (99.1)	1 (0.9)	12
*A. flavus*	93	28 (30.1)	52 (55.9)	13 (14.0)	27 (29.0)	51 (54.8)	12 (12.9)	90 (96.8)	3 (3.3)	7
*A. niger*	75	40 (53.3)	22 (29.3)	13 (17.4)	40 (53.3)	22 (29.3)	11 (14.7)	73 (97.3)	2 (2.7)	12
*A. terreus*	43	12 (27.9)	16 (37.2)	15 (34.9)	12 (27.9)	16 (37.2)	15 (34.9)	43 (100)	0	10
*A. versicolor*	11	7 (63.6)	2 (18.2)	2 (18.2)	7 (63.6)	2 (18.2)	1 (9.1)	10 (90.9)	1 (9.1)	10
*A. sydowii*	11	1 (9.0)	5 (45.5)	5 (45.5)	0	0	0	0	11 (100)	1
*A. nidulans*	10	6 (60.0)	3 (30.0)	1 (10.0)	6 (60.0)	3 (30.0)	1 (10.0)	10 (100)	0	9
Subtotal	350	112 (32.0)	180 (51.4)	58 (16.6)	110 (31.4)	174 (49.7)	48 (13.7)	332 (94.8)	18 (5.2)	61
***ASPERGILLUS*** **SPECIES RARELY ENCOUNTERED (*****n*** < **10)**										
*A. tubingensis*	7	1 (14.2)	3 (42.9)	3 (42.9)	0	0	0	0	7 (100)	0
*A. japonicus*^a^	5	0	0	5 (100)	0	0	0	0	4 (80.0)	0
*A. nomius*	4	0	1 (25.0)	3 (75.0)	0	1 (25.0)	2 (50.0)	3 (75.0)	1 (25.0)	1
*A. tamarri*	3	0	1 (33.3)	2 (66.7)	0	1 (33.3)	0	1 (33.3)	2 (66.7)	1
*A. aculeatus*	2	0	0	2 (100)	0	0	0	0	2 (100)	0
*A. ustus*	2	1 (50.0)	1 (50.0)	0	1 (50.0)	0	1 (50.0)	2 (100)	0	2
Other species^b^	8	1 (12.5)	2 (25.0)	5 (62.5)	1 (12.5)	0	0	1 (12.5)	7 (87.5)	6^c^
Subtotal	31	3 (9.7)	8 (25.8)	20 (64.5)	2 (6.5)	2 (6.5)	3 (9.6)	7 (22.6)	24 (77.4)	10
Total	381	115 (30.2)	188 (49.3)	78 (20.5)	112 (29.4)	176 (46.2)	51 (13.4)	339 (89.0)	41 (10.8)	71

a *One isolate of A. japonicas was identified as Penicillum olsonii with identification score of 1.330*.

b *Includes one each of A. oryzae, A. cristatus, A. turcosus, A. caesiellus, A. austroafricanus, A. quadrilineatus, A. unguis, and A. luchuensis*.

c *Among these eight species, reference spectra in the Bruker Filamentous library 1.0 are present only for A. unguis (two reference spectra) and A. oryzae (four reference spectra)*.

### Identification by molecular methods

ITS region sequencing analysis was performed as previously described to identify all the *Aspergillus* isolates (Ciardo et al., 2007, 2010). β-tubulin gene sequencing analysis was carried out for species level identification to those *Aspergillus* isolates verified by ITS gene primarily. If the species level results of the β-tubulin sequencing analysis were not acceptable, then the calmodulin gene was sequenced additionally (Tam et al., 2014). Sequence alignment and assignment to species/genus level was done according to guidelines published previously (Ciardo et al., 2007, 2010). The results of ITS, β-tubulin, and calmodulin sequencing analyses were considered acceptable if homology with other entries in the databases was >98%. The results were also considered not acceptable if two species each with a probability of >98% were identified but the difference in identification probability was < 0.8% (Schulthess et al., 2014).

### Identification by the Bruker Biotyper MALDI-TOF MS system

Preparation of *Aspergillus* isolates for MALDI-TOF MS identification was done according to the manufacturer's instructions with some modifications (i.e., sub-cultivation of *Aspergillus* isolates from solid media to liquid media was not done and harvest of more outer mycelia from the colonies after prolonged incubation was performed). Isolates for MALDI-TOF MS analysis were cultivated on Sabouraud Dextrose Agar (SDA) plates (Becton Dickinson Microbiology Systems, Sparks, MD, USA) under aerobic conditions at 28°C for 2–5 days depending on the growth rate of the colony. The majority of these isolates were analyzed by the Bruker Biotyper MALDI-TOF MS system after 48 h of growth on SDA plates, although a few isolates were analyzed when the diameter of the colonies reached 1–2 cm after 24–120 h of incubation. The outer mycelia of the colony were collected with a sterile inoculating loop and then transferred to 1.5-mL Eppendorf tube containing 300 μl distilled water and 900 μl ethanol. The suspension was centrifuged at 13,000 rpm for 3 min, and the pellet was dried at room temperature (RT) for 10–30 min. The pellet was then re-suspended in 50–80 μl of 70% formic acid. After an incubation of 10 min at RT, an equal volume of acetonitrile was added. Samples were incubated again at RT for 10 min and subsequently centrifuged at 13,000 rpm for 2 min. Supernatant (1 μl) was transferred to a polished steel MSP 96 target plate (Bruker Daltonik) and allowed to dry at RT before being overlaid with 1 μl of matrix solution (Bruker Daltonik).

The acquisition and analysis of mass spectra were performed by a Bruker Microflex™ LT mass spectrometer (Bruker Daltonik GmbH) at NTUH and by an Autoflex™ LT mass spectrometer (Bruker Daltonik GmbH) at PUMCH using the Bruker Biotyper MALDI MS software package (version 3.1) with the Filamentous Fungi Library 1.0 (Bruker Daltonik). The Bruker bacterial test standard (Bruker Daltonik) was used for calibration according to the instructions of the manufacturer. Identification scores of ≥2.000 indicated species-level identification, scores of 1.700–1.999 indicated genus-level identification, and scores of <1.700 were considered unreliable. Isolates with unreliable scores were reanalyzed using the same procedure.

We also evaluated the performance of the Bruker Biotyper MALDI system for *Aspergillus* identification by lowering the species-level identification cutoff score values from ≥2.000 to ≥1.900, ≥1.800, and ≥1.700 and the genus-level identification cutoff score values from 1.700–1.999 to 1.600–1.899, 1.500–1.799, and 1.400–1.699 followed by reinterpreting the top 10 matching database records.

## Results

### Identification of *Aspergillus* isolates by molecular methods

Among the 381 isolates of *Aspergillus*, 21 different species were identified by the molecular methods (Table 1). All the tested isolates were successfully sequenced by ITS region to *Aspergillus* genus or certain species complex level. The majority (*n* = 282, 74.0%) of these isolates were identified to the species level by β-tubulin gene sequencing analysis except for *A. flavi* complex isolates (*n* = 94, 24.7%) with confused alignment results between *Aspergillus flavus* and *A. oryzae* primarily, A little part of *A. nigri* complex isolates (*n* = 3, 0.8%) and some uncommon *Aspergillus* species isolates (*n* = 2, 0.5%) were also failed to be identified by β-tubulin gene. Calmodulin gene sequencing was carried out further to identify those remained isolates with ambiguous species information (*n* = 99, 26.0%) and performed particularly well in identification of *A. flavi* complex member species than β-tubulin gene in this study which should be recommended as a first-line sequencing gene for *A. flavi* complex in the future. Isolates of *Aspergillus* species numbering ≥10 were arbitrarily defined as commonly encountered species. These species (*n* = 350, 91.9%) included *A. fumigatus, A. flavus, A. niger, A. terreus, A. versicolor, A. sydowii*, and *A. nidulans* (Table 1). The remaining 14 *Aspergillus* species (*n* = 31, 8.1%) were classified as rarely encountered isolates.

### Identification of *Aspergillus* isolates by Bruker Biotyper MALDI-TOF MS

Applying the standard interpretative criteria recommended by the manufacturer, i.e., a species cutoff value of ≥2.000 and a genus cutoff value of 1.700–1.999, the Bruker MALDI-TOF system identified 115 (30.2%) of the 381 isolates to the species level and 188 (49.3%) isolates to the genus level. A total of 78 (20.5%) isolates had score values of ≤ 1.699 and were, therefore, not identifiable (Table 1). The ability of the Bruker Biotyper MALDI-TOF MS system for identifying isolates to the species level (score values of ≥2.000) was much higher for commonly encountered *Aspergillus* species (*A. fumigatus, A. flavus, A. niger, A. terreus, A. versicolor, A. sydowii*, and *A. nidulans*; *n* = 110, 31.4% [110/350]) than for rare *Aspergillus* species (*n* = 2, 6.5% [2/31]). Comparison of the identification results of isolates with score values of ≥2.000 with those obtained by the molecular methods revealed that one isolate of *A. flavus* was misidentified as *A. oryzae*, one isolate of *A. sydowii* was incorrectly identified as *A. versicolor* and one isolate of *A. tubingensis* was erroneously identified as *A. niger* (Table S1).

A correct species-level identification rate of 89.0% (339/381) could be reached by the Bruker Biotyper MALDI-TOF MS system regardless of the score values obtained. Among the 339 isolates, 85.0% (288/339) isolates had the scores of ≥1.700. Forty-one isolates (10.8%) achieved only accurate genus identification as *Aspergillus* species and most of them had lower score values of ≤ 1.699. After lowering the species-level identification cutoff values from ≥2.000 to ≥1.900, ≥1.800, and ≥1.700, the species-level identification rates increased significantly from 30.2% (115/381) at a cutoff of ≥2.000 to 46.5% (177/381) at a cutoff of ≥1.900, 62.0% (236/381) at a cutoff of ≥1.800 and 79.5% (303/381) at a cutoff of ≥1.700. The adjusted criteria produced a slight variation in misidentification rates at the species level, ranged from 0.8% (3/381) at a cutoff of ≥2.000 to 2.1% (8/381) at a cutoff of ≥1.900, 2.4% (9/391) at a cutoff of ≥1.800 and 3.9% (15/381) at a cutoff of ≥1.700. The misidentified species were *A. flavus, A. sydowii*, and *A. tubingensis*. Adjustment of the genus cutoff value from 1.700–1.999 to 1.600–1.899, 1.500–1.799, and 1.400–1.699 did not result in additional genus-level misidentification, although the correct genus-level identification rate decreased from 49.3% (188/381) to 38.6% (147/381), 27.8% (106/381), and 13.9% (53/381), respectively, because the majority of the isolates were reclassified to the species level (Table 2).

**Table 2:** Re-evaluation of the Bruker MALDI-TOF results with adjusted cutoff values.

***Aspergillus* species**	**No. of isolates**	**No. (%) of isolates with indicated score values identified by the Bruker Biotyper MALDI-TOF MS system**															
		**Identification with adjusted species level cutoff values in the reference of molecular results**								**Identification with adjusted genus level cutoff values in the reference of molecular results**							
		**≥2.000**		**≥1.900**		**≥1.800**		**≥1.700**		**1.700–1.999**		**1.600–1.899**		**1.500–1.799**		**1.400–1.699**	
		**C**	**IC**	**C**	**IC**	**C**	**IC**	**C**	**IC**	**C**	**IC**	**C**	**IC**	**C**	**IC**	**C**	**IC**
*A. fumigatus*	107	18 (16.8)	0	44 (41.1)	0	74 (69.2)	0	98 (91.6)	0	80 (74.8)	0	57 (53.3)	0	29 (27.1)	0	6 (5.6)	0
*A. flavus*	93	27 (29.0)	1 (1.1)	42 (45.2)	2 (2.2)	56 (60.2)	2 (2.2)	78 (83.9)	2 (2.2)	52 (55.9)	0	40 (43.0)	0	33 (35.5)	0	12 (12.9)	0
*A. niger*	75	40 (53.3)	0	50 (66.7)	0	57 (76.0)	0	62 (82.7)	0	22 (29.3)	0	16 (21.3)	0	11 (14.7)	0	8 (10.7)	0
*A. terreus*	43	12 (27.9)	0	17 (39.5)	0	20 (46.5)	0	28 (65.1)	0	16 (37.2)	0	17 (39.5)	0	16 (37.2)	0	11 (25.6)	0
*A. versicolor*	11	7 (63.6)	0	7 (63.6)	0	8 (72.7)	0	9 (81.8)	0	2 (18.2)	0	4 (36.4)	0	3 (27.3)	0	2 (18.2)	0
*A. sydowii*	11	0	1 (9.1)	0	3 (27.3)	0	3 (27.3)	0	6 (54.6)	5 (45.5)	0	3 (27.3)	0	5 (45.4)	0	3 (27.3)	0
*A. nidulans*	10	6 (60.0)	0	7 (70.0)	0	9 (90.0)	0	9 (90.0)	0	3 (30.0)	0	3 (30.0)	0	1 (10.0)	0	1 (10.0)	0
*A. tubingensis*	7	0	1 (14.3)	0	2 (28.6)	0	4 (57.2)	0	5 (71.4)	4 (57.2)	0	3 (42.9)	0	1 (14.3)	0	1 (14.3)	0
*A. japonicus*	5	0	0	0	0	0	0	0	0	0	0	1 (20.0)	0	1 (20.0)	0	2 (40.0)	0
*A. nomius*	4	0	0	0	0	0	0	1 (25.0)	0	1 (25.0)	0	1 (25.0)	0	4 (100)	0	1	0
*A. tamarri*	3	0	0	0	0	1 (33.3)	0	1 (33.3)	0	1 (33.3)	0	1 (33.3)	0	0	0	2	0
*A. aculeatus*	2	0	0	0	0	0	0	0	0	0	0	0	0	0	0	0	0
*A. ustus*	2	1 (50.0)	0	1 (50.0)	0	1 (50.0)	0	1 (50.0)	0	0	0	0	0	0	0	1	0
Others	8	1 (12.5)	0	1 (12.5)	1 (12.5)	0	1 (12.5)	1 (12.5)	2 (25.0)	2 (25.0)	0	1 (12.5)	0	2 (25.0)	0	3	0
Total (%)	381 (100)	112 (29.4)	3 (0.8)	169 (44.4)	8 (2.1)	227 (59.6)	9 (2.4)	288 (75.6)	15 (3.9)	188 (49.3)	0	147 (38.6)	0	106 (27.8)	0	53 (13.9)	0

*C, correct identification; IC, incorrect identification*.

The Bruker Biotyper MALDI-TOF MS system did not misidentify any of the isolates of *A. fumigatus, A. niger, A. terreus, A. nidulans*, or *A. versicolor* regardless of the score values obtained. Two *A. flavus* isolates were identified as *A. oryzae* (the first best match) and *A. flavus* (the second best match) with differences in score values of < 0.06. All of the 11 *A. sydowii* isolates were misidentified as *A. versicolor* with scores ranging from 2.046 to 1.081, although only one reference spectrum of *A. sydowii* was included in the database. The rare *Aspergillus* species isolates, namely *A. japonicus, A. tubingensis*, and *A. aculeatus*, were all misidentified, most likely because of the lack of reference spectra in the database. The Bruker Biotyper MALDI-TOF MS system resulted in the misidentification of one isolate of *A. japonicus* as *Penicillium olsonii* (score value, 1.330; Table S1).

### Comparison of results by conventional identification methods based on morphological characteristics with those by Bruker Biotyper MALDI-TOF MS system

The conventional phenotypic identification methods identified 96.3% (367/381) of the isolates to the species level and the rest to the genus level. Molecular analysis revealed that 89.6% (329/367) of the isolates had been correctly identified and 10.4% (38/367) had been incorrectly identified by conventional phenotypic identification methods. In comparison, using a cutoff score of ≥2.000, the Bruker Biotyper MALDI-TOF MS system identified 30.2% (115/381) of the tested isolates to the species level; however, 2.6% (3/115) of those isolates were misidentified. Using the adjusted cutoff value of ≥1.700, the species-level identification rate increased to 79.5% (303/381) and the misidentification rate increased to 5.0% (15/303). Although, the species-level identification rates obtained by the Bruker Biotyper MALDI-TOF MS system were lower than the rate obtained by the conventional phenotypic identification method (30.2% and 79.5 vs. 96.3%), the Bruker Biotyper MALDI-TOF MS system resulted in higher rates of accurate species-level identification than the conventional method (97.4%, 95.0 vs. 89.6%) and lower rates of misidentification (2.6%, 5.0 vs. 10.4%; Table 3). For rare *Aspergillus* species, however, the rates of accurate species-level identification were markedly lower for conventional methods and the Bruker Biotyper MALDI-TOF MS system than for the molecular methods of identification (Table 3).

**Table 3:** Comparison of identification results of 381 isolates of *Aspergillus* to species level by the routine morphological identification and those by the Bruker Biotyper MALDI-TOF MS system with different score values.

***Aspergillus* species**	**No. of isolates**	**No. of isolates with indicated identification result/no. of isolates (%) obtained from conventional morphological methods and the Bruker Biotyper MALDI-TOF MS system with indicated ranges of score values**							
		**Identification to species level by conventional morphological methods (*****n*** = **367)**		**No. of isolates with indicated score values obtained by the Bruker Biotyper MALDI-TOF MS system**			
		**Correct identification**	**Incorrect identification**	**≥2.000 (*****n*** = **115)**		**≥1.700 (*****n*** = **303)**	
				**Correct identification**	**Incorrect identification**	**Correct identification**	**Incorrect identification**
***ASPERGILLUS*** **SPECIES COMMONLY ENCOUNTERED (*****n*** **≥ 10)**									
*A. fumigatus*	107	105/105 (100)	0	18 /18 (100)	0	98/98 (100)	0
*A. flavus*	93	91/91 (100)	0	27/28 (96.4)	1/28 (3.6)	78/80 (97.5)	2/80 (2.5)
*A. niger*	75	74/75 (98.7)	1/75 (1.3)	40/40 (100)	0	62/62 (100)	0
*A. terreus*	43	42/42 (100)	0	12/12 (100)	0	28/28 (100)	0
*A. versicolor*	11	2/11 (18.2)	9/11 (81.8)	7/7 (100)	0	9/9 (100)	0
*A. sydowii*	11	6/6 (100)	0	0	1/1 (100)	0	6/6 (100)
*A. nidulans*	10	9/10 (90.0)	1/10 (10.0)	6/6 (100)	0	9/9 (100)	0
Subtotal	350	329/340 (96.8)	11/340 (3.2)	110/112 (98.2)	2/112 (1.8)	284/292 (97.3)	8/292 (2.7)
***ASPERGILLUS*** **SPECIES RARELY ENCOUNTERED (*****n*** < **10)**									
*A. tubingensis*	7	0	7/7 (100)	0	1/1 (100)	0	5/5 (100)
*A. japonicus*	5	0	5/5 (100)	0	0	0	0
*A. nomius*	4	0	4/4 (100)	0	0	1/1 (100)	0
*A. tamarri*	3	0	2/2 (100)	0	0	1/1 (100)	0
*A. aculeatus*	2	0	2/2 (100)	0	0	0	0
*A. ustus*	2	0	0	1/1 (100)	0	1/1 (100)	0
Others	8	0	7/7 (100)	1/1 (100)	0	1/3 (33.3)	2/3 (66.7)
Subtotal	31	0	27/27 (100)	2/3 (66.7)	1/3 (33.3)	4/11 (36.4)	7/11 (63.6)
Total (%)	381	329/367 (89.6)	38/367 (10.4)	112 /115 (97.4)	3/115 (2.6)	288/303 (95.0)	15/303 (5.0)

## Discussion

MALDI-TOF MS has increasingly been employed in microbiology laboratories to rapidly and accurately identify clinical pathogenic bacteria and yeasts (Ling et al., 2014; Singhal et al., 2015). In July 2012 Bruker Daltonic released the first version of the filamentous fungi library, making mold identification by MALDI-TOF MS promising. However, few definitive conclusions about the practicability of MALDI-TOF for mold identification have been drawn since the release of the database even though much research has been conducted, especially for *Aspergillus*, the most important filamentous fungal pathogen encountered in clinical laboratories (Alanio et al., 2011; De Carolis et al., 2012; Lau et al., 2013; Ranque et al., 2014; Schulthess et al., 2014). This may be due partially to the complexity of *Aspergillus* cell structure and phylogeny (Samson et al., 2014), insufficient fungal coverage in the database, the impracticability of liquid culturing of molds as recommended by the manufacturer and the limitations of some user in-house databases (Lau et al., 2013; Chen et al., 2015).

In this study we evaluated the performance of the Bruker Biotyper MALDI-TOF MS system using the first commercial Filamentous Fungi database developed by Bruker Daltonik for the identification of clinical *Aspergillus* cultured on solid media. Although, the Bruker system recommends liquid cultivation of filamentous fungi, we decided to inoculate our *Aspergillus* isolates on solid media for two main reasons: (i) liquid cultivation of *Aspergillus* is rarely used in clinical mycology laboratories because of the risk for aerosolized spore contamination and the inability to visualize phenotypic macro- and microscopic characteristics (Lau et al., 2013); in addition, liquid cultivation makes it difficult to detect contamination or co-infection with other molds; (ii) solid cultivation of *Aspergillus* is not only employed extensively in clinical mycology laboratories but also makes it easy to distinguish contamination. Collecting fungal material directly from solid medium instead of harvesting it from liquid subculture could simplify the sample preparation and save time (Lau et al., 2013; Cassagne et al., 2016).

A total of 381 well-characterized *Aspergillus* isolates comprising of 21 species were collected for analysis. Using the manufacturer's recommended cutoff values for species and genus identification, we found that the Bruker Biotyper MALDI-TOF MS system could only identify 30.2% of the isolates to the species level (cutoff score ≥2.000) and 49.3% to the genus level (cutoff scores 1.700–1.999). In addition, 20.5% of the isolates could not be reliably identified (scores < 1.700). There are a number of possible reasons to explain the poor performance of MALDI-TOF for *Aspergillus* identification with manufacturer-recommended interpretation criteria. First, the database lacks good fungal coverage. Only 90 reference spectra representing 19 *Aspergillus* species are included in the Bruker filamentous fungi library 1.0 and few of the unusual *Aspergillus* species are presented. Second, the method of culturing *Aspergillus* isolates may affect the identification results to some degree. Mycelia are the main products when isolates are cultured in liquid media as recommended by the manufacturer; however, solid cultivation allows for the harvest of spores and mycelia, which have different biological phases and MALDI-TOF spectra (Alanio et al., 2011; Samson et al., 2014). In order to eliminate the discrepancy between those two materials about their suitability for the database, almost the *Aspergillus* isolates tested in this study were incubated on solid agar media for 48 h and outer part (mainly mycelia materials) of the grown colonies were harvested for MALDI-TOF MS analysis. This modified and reliable method is important for clinical mycology laboratories because it is time-saving for identification of *Aspergillus* species on 2 days instead of 5–14 days with conventional processing. Third, the species-level identification score values of ≥2.000 recommended by the manufacturer may be too high for filamentous fungi although it is appropriate for the identification of bacteria (Bilecen et al., 2015; Singhal et al., 2015). Of the 188 isolates which could only be identified to the genus level (scores of 1.700–1.999) by MALDI-TOF, 176 (93.6%) were correctly identified at the species level according to the molecular results. Furthermore, of the 78 isolates that could not be identified (scores < 1.700), 51 (65.4%) were correctly identified to the species level and 26 (33.3%) were correctly identified to the genus level. Only one isolate of *A. japonicus* was misidentified as *P. olsonii* (score, 1.330). Based on those findings, we intended to re-evaluate the performance of the Bruker Biotyper MALDI-TOF MS system for *Aspergillus* identification with lower adjusted criteria.

After lowering the species-level cutoff value from ≥2.000 to ≥1.700, the rate of species-level identification increased markedly from 30.2 to 79.5%. The error rate, however, also increased, from 2.6 to 5.0%. Lowering the genus-level cutoff value from 1.700–1.999 to 1.400–1.699 did not result in any genus-level misidentifications.

Further analysis in this study, we found that the Bruker MALDI-TOF MS system with our adjusted cutoff value criteria had a preferable performance in identification of common *Aspergillus* like *A. fumigatus, A. flavus, A. niger, A. nidulans, A. terreus*, and *A. versicolor* to the species level (rates ~80%). For *A. sydowii*, all isolates (*n* = 11) were misidentified as *A. versicolor*, although one *A. sydowii* reference spectrum was included in the database. The incapable identification to *A. sydowii* by Bruker Biotyper should be attributed to insufficient spectra in the library and the bad representativeness of this spectrum listed, which suggests that the Bruker Biotyper MALDI-TOF MS system currently is not reliable for *A. sydowii* identification. As for the uncommon *Aspergillus* species, the poor performance of the Bruker MALDI-TOF MS might be attributed to the absence of spectra in the database principally.

In this study, Bruker Biotyper did not perform much better than morphological method in *Aspergillus* identification, particularly for common *Aspergillus* species. However, as the emergence of some uncommon *Aspergillus* species infection in the clinical and some common *Aspergillus* isolates could be mutated in phenotype (less sporulation, slow growth, etc.) under the pressure of antifungals treatment and host immune defense, we firmly believe that Bruker Biotyper MALDI-TOF MS can preform superior than morphology in *Aspergillus* identification in the future, especially after continued enlarging the spectra library.

## Conclusion

The Bruker Biotyper MALDI-TOF MS system, when used in conjunction with the Filamentous Fungi Library 1.0, is a promising method for the identification of *Aspergillus* directly cultured on solid media. However, at present, the system should not fully replace conventional identification methods. Expansion of the database and adoption of appropriate cutoff values are essential to improve the capacity of the Bruker Biotyper MALDI-TOF MS system for identification of *Aspergillus* species.

## Author contributions

YL, HW, YX, and PH designed the experiments, performed the experiments, analyzed the data, and participated in the writing of the manuscript. YL, HW, YZ, YX, and PH read and approved the final version of the manuscript.

### Conflict of interest statement

The authors declare that the research was conducted in the absence of any commercial or financial relationships that could be construed as a potential conflict of interest.
