# Supplementary material for: Evaluation of the Bruker Biotyper Matrix-Assisted Laser Desorption/Ionization Time-of-Flight Mass Spectrometry System for Identification of Aspergillus Species Directly from Growth on Solid Agar Media
Source: Front Microbiol. 2017 Jun 29;8:1209. doi: 10.3389/fmicb.2017.01209 (PMC5489701; doi:10.3389/fmicb.2017.01209)
Supplement: Supplementary file 1 [file Table1.doc]

**Table S1. Discrepant identification results by gene sequencing analysis, the Bruker Biotyper MALDI-TOF MS system, and conventional morphological identification methods**

| **Gene sequencing analysis** | | **Morphological identification results** | **Identification results by the MALDI-TOF MS system** | | | |  |
| --- | --- | --- | --- | --- | --- | --- | --- |
| Identification results  (Identity %) | Accession No.  of β-tubulin gene |  | Best match | Score value | Second best match | Score value |  |
| ***A. flavus* (100)** | | **HF570048.1*** | ***A. flavus*** | ***A. oryzae*** | **2.077** | ***A. flavus*** | **2.060** |
| *A. flavus* (100) | | EF661505.1* | *A. flavus* | *A. oryzae* | 1.995 | *A. flavus* | 1.946 |
| *A. niger* (100) | | EF661089.1 | *A. niger* | *A. nidulans* | 1.305 | *A. candidus* | 1.301 |
| *A. niger* (100) | | KC175288.1 | *A. niger* | *A. candidus* | 1.619 | *A. niger* | 1.438 |
| *A. nomius* (99.8) | | AY017582.1 | *A. flavus* | *[A. flavus](mhtml:file://D:\\倫\\MALDI TOF\\Aspergillus species\\MALDI try extraction\\asp1124.mht!file:///C:\\Users\\tof-user\\AppData\\Roaming\\Bruker Daltonik\\MALDIBiotyperAutomationControl\\HtmpResults\\20151124 NTUH Fungi repeat.html)* | 1.505 | *[A. flavus](mhtml:file://D:\\倫\\MALDI TOF\\Aspergillus species\\MALDI try extraction\\asp1124.mht!file:///C:\\Users\\tof-user\\AppData\\Roaming\\Bruker Daltonik\\MALDIBiotyperAutomationControl\\HtmpResults\\20151124 NTUH Fungi repeat.html)* | 1.403 |
| *A. nomius* (99.3) | | EF661494.1 | *A. flavus* | *A. nomius* | 1.540 | *A. nomius* | 1.307 |
| *A. nomius* (99.3) | | AY017582.1 | *A. flavus* | *A. nomius* | 1.542 | *A. nomius* | 1.474 |
| *A. sydowii* (100) | | EF428373.1 | *A. sydowii* | *A. versicolor* | 1.569 | *A. versicolor* | 1.349 |
| *A. sydowii* (100) | | EF428373.1 | *A. sydowii* | *A. versicolor* | 1.598 | *A. versicolor* | 1.596 |
| *A. sydowii* (100) | | EF428373.1 | *A. sydowii* | *A. versicolor* | 1.714 | *A. versicolor* | 1.633 |
| *A. sydowii* (100) | | EF428373.1 | *A. sydowii* | *A. versicolor* | 1.900 | *A. versicolor* | 1.875 |
| *A. sydowii* (100) | | EF428373.1 | *A. sydowii* | *A. versicolor* | 1.081 | *A. versicolor* | 1.076 |
| *A. sydowii* (99.8) | | KT427381.1 | *A. sydowii* | *A. versicolor* | 1.484 | *A. versicolor* | 1.410 |
| *A. sydowii* (100) | | EF428373.1 | *Aspergillus* spp. | *A. versicolor* | 1.911 | *A. versicolor* | 1.808 |
| *A. sydowii* (100) | | EF428373.1 | *Aspergillus* spp. | *A. versicolor* | 1.787 | *A. versicolor* | 1.526 |
| ***A. sydowii* (100)** | | **EF428373.1** | ***Aspergillus* spp.** | ***A. versicolor*** | **2.046** | ***A. versicolor*** | **1.888** |
| *A. sydowii* (99.6) | | KT427381.1 | *A. versicolor* | *A. versicolor* | 1.380 | *Penicillum chrysogenum* | 1.290 |
| *A. sydowii* (100) | | KT427381.1 | *Aspergillus* spp. | *A. versicolor* | 1.782 | *A. versicolor* | 1.452 |
| *A. tubingensis* (100) | | HQ632664.1 | *A. niger* | *A. niger* | 1.457 | *Penicillum turbatum* | 1.381 |
| *A. tubingensis* (99.4) | | KJ136082.1 | *A. niger* | *A. niger* | 1.700 | *A. niger* | 1.642 |
| *A. tubingensis* (99.7) | | KJ136082.1 | *A. niger* | *A. niger* | 1.856 | *A. niger* | 1.845 |
| *A. tubingensis* (100) | | HQ632664.1 | *A. niger* | *A. niger* | 1.946 | *A. niger* | 1.893 |
| ***A. tubingensis* (100)** | | **HQ632707.1** | ***A. niger*** | ***A. niger*** | **2.041** | ***A. niger*** | **1.974** |
| *A. tubingensis* (100) | | HQ632707.1 | *A. niger* | *A. niger* | 1.817 | *A. niger* | 1.803 |
| *A. tubingensis* (99.5) | | KJ136082.1 | *A. niger* | *A. niger* | 1.265 | *A. niger* | 1.216 |
| *A. japonicus* (99.8) | | EF661143.1* | *A. niger* | *A. niger* | 1.468 | *A. niger* | 1.445 |
| *A. japonicus* (99.8) | | EF661143.1* | *A. niger* | *A. niger* | 1.384 | *A. niger* | 1.361 |
| *A. japonicus* (99.7) | | EF661143.1* | *A. niger* | *[A. niger](mhtml:file://D:\\倫\\MALDI TOF\\Aspergillus species\\MALDI try extraction\\1120-fungus2.mht!file:///C:\\Users\\tof-user\\AppData\\Roaming\\Bruker Daltonik\\MALDIBiotyperAutomationControl\\HtmpResults\\20151118 NTUH Fungi.html)* | 1.682 | *[A. niger](mhtml:file://D:\\倫\\MALDI TOF\\Aspergillus species\\MALDI try extraction\\1120-fungus2.mht!file:///C:\\Users\\tof-user\\AppData\\Roaming\\Bruker Daltonik\\MALDIBiotyperAutomationControl\\HtmpResults\\20151118 NTUH Fungi.html)* | 1.654 |
| *A. japonicus* (98.9) | | AY820017.1 | *A. niger* | *A. terreus* | 1.332 | *Penicillum citreonigrum* | 1.192 |
| *A. japonicas* (98.5) | | AY820017.1 | *A. niger* | *Penicillum olsonii* | 1.330 | *A. sydowii* | 1.202 |
| *A. tamarii* (100) | | AY017540.1 | *A. flavus* | *[A. flavus](mhtml:file://D:\\倫\\MALDI TOF\\Aspergillus species\\MALDI try extraction\\asp1124.mht!file:///C:\\Users\\tof-user\\AppData\\Roaming\\Bruker Daltonik\\MALDIBiotyperAutomationControl\\HtmpResults\\20151124 NTUH Fungi repeat.html)* | 1.418 | *[A. flavus](mhtml:file://D:\\倫\\MALDI TOF\\Aspergillus species\\MALDI try extraction\\asp1124.mht!file:///C:\\Users\\tof-user\\AppData\\Roaming\\Bruker Daltonik\\MALDIBiotyperAutomationControl\\HtmpResults\\20151124 NTUH Fungi repeat.html)* | 1.396 |
| *A. tamarii* (100) | | AY017540.1 | *A. flavus* | *A. oryzae* | 1.430 | *A. fumigatus* | 1.321 |
| *A. aculeatus* (99.3) | | KU310906.1 | *A. niger* | *A. niger* | 1.243 | *A. niger* | 1.222 |
| *A. aculeatus* (99.5) | | KU310906.1 | *A. niger* | *A. niger* | 1.276 | *A. niger* | 1.223 |
| *A. cristatus* (99.1) | | FJ608411.1 | *A. glaucus* | *A. fumigatus* | 1.331 | *A. fumigatus* | 1.199 |
| *A. turcosus* (99.7) | | LN874003.1 | *A. fumigatus* | *A. fumigatus* | 1.493 | *A. glaucus* | 1.286 |
| *A. caesiellus* (99.4) | | EF651884.1 | *A. sydowii* | *A. versicolor* | 1.931 | *A. versicolor* | 1.788 |
| *A. austroafricanus* (99.8) | | JN854025.1* | *Aspergillus* spp. | *A. nidulans* | 1.435 | *A. nidulans* | 1.332 |
| *A. quadrilineatus* (99.1) | | EF652317.1 | *A. nidulans* | *A. nidulans* | 1.772 | *A. nidulans* | 1.698 |
| *A. unguis* (99.2) | | EF652333.1 | *A. versicolor* | *A. versicolor* | 1.570 | *A. versicolor* | 1.513 |
| *A. luchuensis* (99.4) | | JX500062.1 | *A. niger* | *A. niger* | 1.390 | *A. candidus* | 1.326 |

**a** *Isolates with identification score values of* ≥*2.000 by Bruker Biotyper MALDI-TOF MS system are shown in boldface.*

**b** *Accession No. with a star mark (*) presented as calmodulin gene ones.*
